# Diversity of the physician workforce: Specialty choice decisions during medical school

**DOI:** 10.1371/journal.pone.0259434

**Published:** 2021-11-04

**Authors:** John Burkhardt, Stephen DesJardins, Larry Gruppen

**Affiliations:** 1 Department of Emergency Medicine and Learning Health Sciences at the University of Michigan Medical School, Ann Arbor, Michigan, United States of America; 2 Center for the Study of Higher and Postsecondary Education at the University of Michigan School of Education and Gerald Ford School of Public Policy, Ann Arbor, Michigan, United States of America; 3 Department of Learning Health Sciences at the University of Michigan Medical School, Ann Arbor, Michigan, United States of America; New Mexico State University, UNITED STATES

## Abstract

**Background:**

Despite efforts to increase the overall diversity of the medical student body, some medical specialties have a less diverse applicant pool based on both gender and race than would be expected based on medical graduate demographics.

**Objectives:**

To identify whether women and Underrepresented in Medicine (URiM) medical students have baseline differences in their career interests or if their career plans change more during medical school when compared to men and non-URIM students.

**Methods:**

Secondary data analyses of all medical students who applied through ERAS from 2005–2010 was conducted. Binary logistic regression models with the response being a planned career in one of four medical specialties (internal medicine, pediatrics, OB/GYN, and general surgery/surgical specialties) at medical school entry and graduation. Regression models included demographics, student attitudes, debt, academic metrics, and medical school experiences.

**Results:**

Comparatively, women were less likely to be interested in internal medicine and surgery and more interested in pediatrics and OB/GYN at matriculation. URiM students expressed more interest in OB/GYN and surgery when starting medical school. At graduation, women were less likely to plan for internal medicine and surgery and were more interested in pursuing OB/GYN and pediatrics. URiM students were more likely to plan for a career in internal medicine and less likely to choose pediatrics.

**Conclusions:**

From matriculation to graduation, women have relatively stable preferences regarding planned medical specialties. In contrast, URiM students’ specialty plans shifted over time among the four specialties, with variation in preferences occurring between matriculation and graduation.

## Introduction

Despite efforts to increase the overall diversity of the medical student body [1–17], some career options [18], such as general surgery [19–23] and the surgical specialties [19, 20, 24–26], obstetrics and gynecology (OB/GYN) [19, 27, 28], pediatrics and its subspecialties [29–31], and emergency medicine (EM) [32–37], have a less diverse applicant pool than would be expected based on medical graduate demographics (by gender, race, or both). These differences have remained largely stable even as women now make up more than 50% of entering medical school classes [38].

Representation matters to patients, not just educators. Physicians treating patients of the same gender [39] or similar racial and ethnic backgrounds may achieve better health outcomes than if these matches are dissimilar [11, 12, 15, 40]. A larger body of evidence reports increased satisfaction from patients with providers from similar backgrounds [41–47] and improved communication with shared-decision making [45, 47–53]. Patient outcome disparities associated with specific racial and ethnic groups in the United States has become more widely reported to the general public during the ongoing COVID pandemic. To ensure equitable care for all patients, now is the time for reevaluating interventions designed to increase physician workforce diversity in all medical specialties.

Many students begin medical school with clear preferences about the specialty area in which they plan to practice [54, 55]. However, as students progress through medical school to graduation the stability of these preferences is less clear [54, 55]. Lifestyle interests, expected income, procedural orientation, societal prestige outside medicine, and the respect of peer physicians within the profession have been correlated with medical specialty selection [56, 57]. Additionally, studies designed to understand how a lack of diversity in the applicant pool persists in EM demonstrated that even when controlling for academic competitiveness, debt, career attitudes and aspirations, women and URiM students had significantly lower interest in that field than their peers [33]. Women were also less likely than men to develop a career interest in EM during medical school and URiM students were less interested in EM at the beginning of medical school and were less likely to maintain or develop an interest in EM compared to their peers [37, 58]. Whereas many academic experiences and opinions may be set prior to medical school, one’s eventual career choice is largely a function of one’s entering career interest and experiences during medical training.

### Objectives

To compare specialty choice associated with gender and race by evaluating two potential mechanisms of underrepresentation: under-recruitment of interest (not convincing people previously uninterested to choose a specific specialty) and failure of interest retention (where individuals change their specialty). The latter could be described as either a passive lack of support for career adoption or a more active, intentional “cooling out” of interest (as described in higher education). “Cooling out” is the “redirection” of a learner’s career aspirations by faculty and advisors [59, 60]. The alternative to “cooling out” is a positive recruitment of students to a new career aspiration, in this case a medical specialty. Our study incorporates these ideas into the definition of recruitment and retention, with the understanding that they are likely providing some contribution to the observed effects. We believe this is an important step toward future policy interventions.

#### Career selection theoretical framework

Concepts from two major applicable theoretical systems, Bounded Rationality Theory (BRT) [61–63] and Bandura’s Theory of Self Efficacy (SE) [64–66], framed this research. Based on BRT, we expect that cognitive limitations and incomplete information availability will drive students to make decisions based on knowable qualities, such as income and specialty competitiveness, instead of acting to maximize their own personal values [61, 62]. SE focuses on one’s belief in his/her ability to influence their cognitive response to adversity and thus persevere toward their goals [64]. Self-efficacy is often enhanced through positive academic experiences and mentorship [64]. Therefore, in addition to individual attitudes previously utilized in career selection studies, an individual’s academic metrics representing likely match competitiveness and a history of positive academic experiences, as well as their level of educational debt are included in the model.

#### Hypotheses

One: At the onset of medical school, compared to men, women are more interested in fields with more female physician role models [67, 68], (pediatrics and OB/GYN) have less interest in fields where women are underrepresented (surgery), and similar interest to men in fields with more gender parity (internal medicine: IM). Two: URiM students will have similar entering interests compared to their non-URiM peers across the four specialty areas examined. Three: Relative to men, women will exhibit evidence of both under-recruitment and lack of interest retention in medical specialties traditionally dominated by males. Four: URiM medical specialty interest will remain relatively stable from entry of medical school to graduation, but will be consistently lower for specialties that have fewer URiM physicians.

## Methods

### Participants

The study uses secondary data of 46,776 students who applied for residency using the Electronic Residency Application Service (ERAS) from 2005 through 2010. Nationally representative datasets from the Association of American Medical Colleges (AAMC), including: Matriculating Student Questionnaire (MSQ), Graduation Questionnaire (GQ) administered after the match, AAMC Applicant Matriculant File (AAMF) were matched to the ERAS to create a longitudinal database. The National Board of Medical Examiners (NBME) U.S. Medical Licensing Exam (USMLE) Step 1 and Step 2 CK score data were also included.

### Variables and outcomes measured

**Fig 1:**
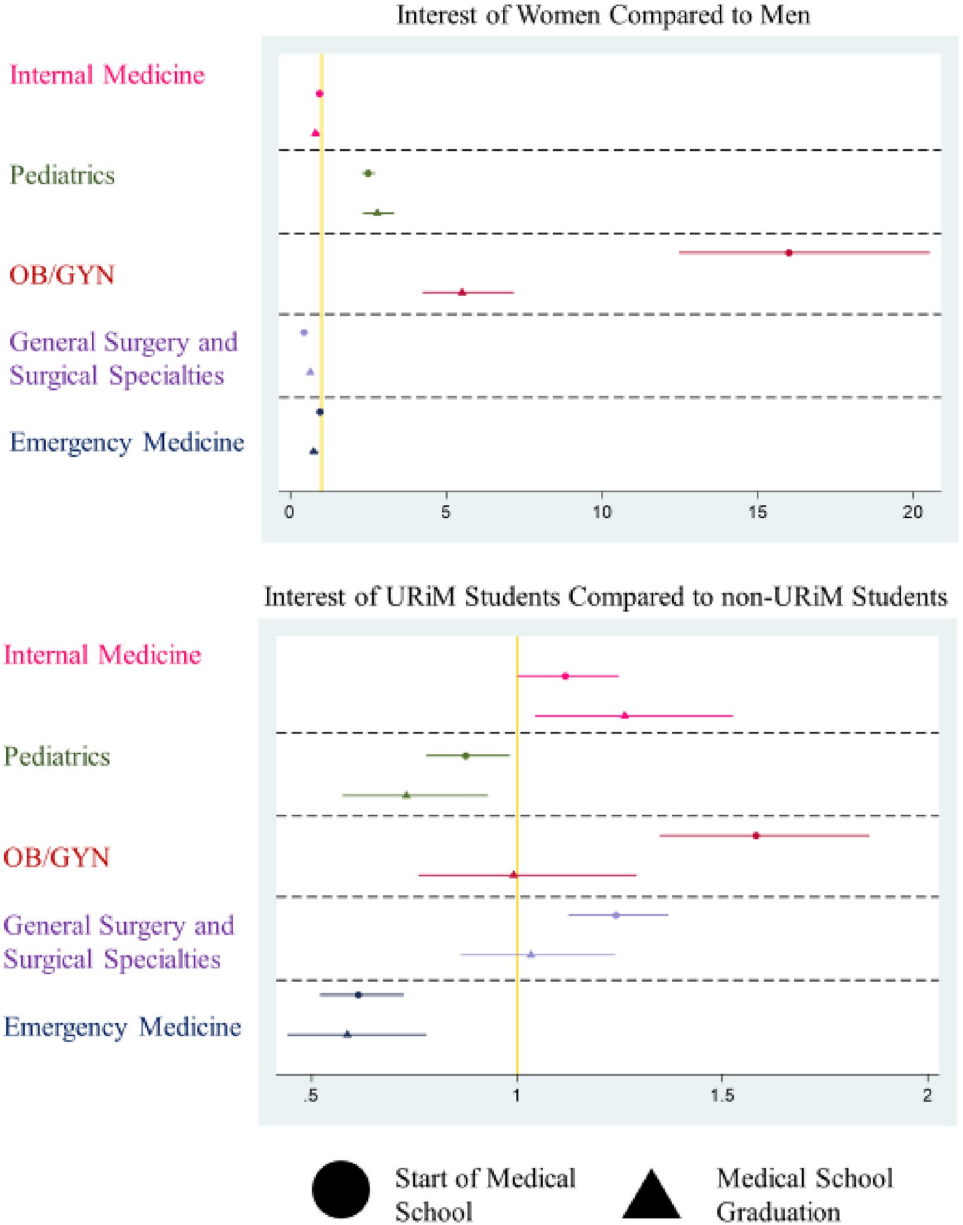
Career interest at onset and graduation. Plot of Odds Ratios from Two Outcomes (Specialty Interest at the Onset of Medical School and Specialty Interest at the time of Graduation) on a logarithmic scale. X-Axis represents the Odds Ratio of the Outcome in each model for each group. Gold line represents Odds of 1 (no effect). Y-Axis represents the medical specialties investigated. Each point represents the calculated Odds Ratio with the 95% Confidence Interval for each value represented by bars to either side.

Predictor variables were selected based on the conceptual framework and the existing literature (Table 1). Students records were only included in the final analysis if they had complete records for all variables included in the final analytical model. No data were imputed. Given the high level of missing data on some of the attitudinal factors recorded on the GQ, a more basic model which only include demographics and USMLE Step Scores was also fitted to provide results with the highest possible number of subjects in addition the model created based on the a priori factors in Table 1. The basic model included total 22,555 subjects. Both models’ results are illustrated in Fig 1 for comparison. The URiM variable represents a binary recoding of a self-reported racial/ethnic identity to be either non-URiM (white or Asian students) or URiM student [69]. Entering career specialty interest was derived from the response to the MSQ item: “What general specialty are you considering?” Graduating career specialty interest was derived from the response to the GQ item: “When thinking about your career, what is your intended area of practice?” Responses were recoded as either “interested in the specialty of study” with all other specialty choices collapsed into “not interested.”

**Table 1:** Variables.

Variable Subgroup Names	Variables within Each Subgroup	Measurement Type	Source(s)
Demographics	Gender	Binary	*Electronic Residency Application Service (ERAS)*
	Age	Continuous	
	URiM Status	Binary	
Entering Attitudes	Opportunity for Authority	Likert-Like Scale	*Matriculating Student Questionnaire (MSQ)*
	Opportunity for Patient Contact		
	Opportunity for Control		
	Opportunity for Decision-Making		
	Opportunity for Expertise in Specialized Area		
	Opportunity to Make a Difference		
	Opportunity for Research		
	Chose Medicine to Limit Stress		
Graduation Attitudes	Work-Life Balance	Likert-Like Scale	*Graduation Questionnaire (GQ)*
	Specialty Competitiveness		
	Specialty Personality		
	Specialty Content		
	Expected Salary		
	Advice from Mentor		
	Family Expectations		
	Debt Level		
Debt Level	Had Pre-Medical Debt	Continuous	*Graduation Questionnaire (GQ)*
	Received Scholarship		
	Medical School Debt in $10,000		
	Non-Educational Debt in $10,000		
Entering GPA	Overall GPA	Continuous	*AAMC Applicant Matriculant File (AAMF)*
	Science GPA		
Standardized Tests	MCAT Total	Continuous	*AAMC Applicant Matriculant File (AAMF)*
	Step 1 Score		
	Step 2 CK Score		*U*.*S*. *Medical Licensing Exam (USMLE)*
Medical School Activity	Number of Publications	Continuous	*Electronic Residency Application Service (ERAS)*
	Research Experience	Continuous	
	Awarded AOA prior to application	Binary	
	Confidence in Specialty Choice	Likert-Like Scale	
	Planned Practice with Underserved Populations		
		Binary	
**Dependent Variables**	Entering Career Interest in IM	Binary	*Matriculating Student Questionnaire (MSQ)*
	Planned Career in IM		
	Entering Career Interest in Peds		
	Planned Career in Peds		
	Entering Career Interest in OB/GYN		*Graduation Questionnaire (GQ)*
	Planned Career in OB/GYN		
	Entering Career Interest in Surgery		
	Planned Career in Surgery		

### Analysis of the outcomes

Eight binary dependent variables were initially examined. Four dependent variables were measures of a plan to enter (or not) a specific career at the beginning of medical school (IM and its specialties, pediatrics and its specialties, OB/GYN, and general surgery and other surgical specialties). These specialties were chosen based on the number of residents in training and because they represented a variety of career options with different patterns of gender and racial representation, including both over and under-representation among the demographic factors included in the study.

The other four dependent variables measured whether the student planned to enter (or not) a specific career (same four specialties) at the time of graduation. Twelve additional models were also fitted by specialty for each of three additional dependent variables: 1) the student planned to enter a specific career (or not) at the time of graduation with the addition of entering interests as a control, 2) a model examining only students who had an entering plan to practice in the same field, and 3) a regression examining only students who did not have an entering plan to practice in the same field. Given the dichotomous nature of all the outcome variables, binary logistic regression was used to fit these models [70] and marginal effects were calculated to provide practical effect size that are easy to interpret. Previously published EM models were included in the figures for further comparison [58].

Institutional Review Board approval was solicited and the study was judged not to require additional regulation or assessment.

## Results

At matriculation, female students were less likely than male students to plan to practice in IM (OR 0.94 95% CI 0.88–0.96) or choose a surgical career (OR 0.42 95% CI 0.40–0.44) (Table 2), but had higher odds of choosing a career in pediatrics than their male peers (OR 2.40 95% CI 2.26–2.54) and OB/GYN (OR 15.19 95% CI 12.73–18.11). At graduation women were also less likely than men to plan for a career in IM (OR 0.81 95% CI 0.70–0.93) or a surgical field (OR 0.64 95% CI 0.56–0.73) and more likely to plan to practice in pediatrics (OR 2.79 95% CI 2.33–3.32) and OB/GYN (OR 5.52 95% CI 4.25–7.18) (Table 3). These results translate to women being 3% less probable to choose IM and 7% less probable to choose a surgical career than men. Conversely, women were 9% more probable to choose pediatrics and 10% more probable to choose OB/GYN than men. Comparisons between medical specialties are displayed in Fig 1 with the two GQ outcome models having qualitatively similar patterns.

**Table 2:** Career interest when entering medical school.

	Internal Medicine (IM)		Pediatrics		OB/GYN		Gen. Surgery and Surgical Specialties	
	Odds Ratio	95% Confidence Interval	Odds Ratio	95% Confidence Interval	Odds Ratio	95% Confidence Interval	Odds Ratio	95% Confidence Interval
Female	0.94*	0.883–0.996	2.40***	2.263–2.549	15.19***	12.73–18.11	0.42***	0.400–0.444
	(0.029)		(0.073)		(1.366)		(0.011)	
Age	1.06***	1.049–1.065	0.92***	0.907–0.927	0.99	0.974–1.004	0.96***	0.952–0.969
	(0.004)		(0.005)		(0.007)		(0.004)	
URiM	1.07	0.989–1.157	0.92*	0.847–0.993	1.60***	1.426–1.794	1.25***	1.166–1.339
	(0.043)		(0.037)		(0.094)		(0.044)	
GPA	1.00	0.999–1.001	1.00*	0.998–1.000	1.00***	0.996–0.999	1.00***	0.997–0.999
	(0.001)		(0.001)		(0.001)		(0.000)	
MCAT	1.03***	1.022–1.037	1.01*	1.000–1.015	1.00	0.985–1.010	1.02***	1.011–1.024
	(0.004)		(0.004)		(0.006)		(0.003)	
Work with Underserved	1.11***	1.059–1.155	1.04	0.999–1.084	1.09*	1.018–1.168	0.62***	0.601–0.645
	(0.024)		(0.022)		(0.038)		(0.011)	
Opportunity for Authority	1.07***	1.045–1.100	0.92***	0.900–0.946	1.06**	1.019–1.109	1.02	0.997–1.041
	(0.014)		(0.012)		(0.023)		(0.011)	
Opportunity for Patient Contact	1.19***	1.147–1.230	1.68***	1.610–1.749	1.29***	1.210–1.378	0.89***	0.866–0.914
	(0.021)		(0.035)		(0.043)		(0.012)	
Opportunity for Control	0.95***	0.916–0.977	0.97*	0.937–0.999	0.92**	0.871–0.973	1.05***	1.020–1.077
	(0.016)		(0.016)		(0.026)		(0.015)	
Opportunity for Decision-Making	0.84***	0.816–0.858	0.87***	0.848–0.889	0.98	0.943–1.021	1.03*	1.003–1.049
	(0.011)		(0.011)		(0.020)		(0.012)	
Opp. for Expert. in Spec. Area	0.97*	0.937–0.995	0.89***	0.861–0.911	1.12***	1.065–1.172	1.32***	1.287–1.362
	(0.015)		(0.013)		(0.027)		(0.019)	
Opportunity to Make a Difference	0.94	0.882–1.009	1.25***	1.139–1.379	1.04	0.886–1.218	0.93*	0.880–0.988
	(0.032)		(0.061)		(0.085)		(0.027)	
Opportunity for Research	1.27***	1.239–1.299	0.96***	0.936–0.981	0.87***	0.836–0.902	1.08***	1.061–1.104
	(0.015)		(0.011)		(0.017)		(0.011)	
Chose Medicine to Limit Stress	0.96*	0.932–0.995	1.05**	1.014–1.079	0.99	0.943–1.047	0.91***	0.883–0.931
	(0.016)		(0.017)		(0.026)		(0.012)	
Constant	0.01***	0.00643–0.0209	0.27***	0.133–0.555	0.01***	0.00292–0.0256	1.80*	1.041–3.122
	(0.003)		(0.099)		(0.005)		(0.505)	
Observations	41,047	41,047	41,047	41,047	41,047	41,047	41,047	41,047

Robust standard errors in parentheses.

*** p<0.001,

** p<0.01,

* p<0.05.

**Table 3:** Career interest when graduating medical school.

	Internal Medicine (IM)		Pediatrics		OB/GYN		Gen. Surgery and Surgical Specialties	
	Odds Ratio	95% Confidence Interval	Odds Ratio	95% Confidence Interval	Odds Ratio	95% Confidence Interval	Odds Ratio	95% Confidence Interval
Female	0.81**	0.697–0.934	2.79***	2.331–3.329	5.52***	4.245–7.167	0.64***	0.561–0.727
	(0.060)		(0.253)		(0.737)		(0.042)	
Age	0.99	0.967–1.018	0.91***	0.875–0.939	0.97	0.929–1.008	1.00	0.975–1.023
	(0.013)		(0.016)		(0.020)		(0.012)	
URiM	1.26*	1.044–1.526	0.73*	0.575–0.928	0.99	0.761–1.292	1.03	0.863–1.239
	(0.122)		(0.089)		(0.134)		(0.095)	
GPA	1.00	0.997–1.003	1.00	0.999–1.006	1.00	0.996–1.004	1.00	0.997–1.002
	(0.001)		(0.002)		(0.002)		(0.001)	
MCAT	1.01	0.987–1.027	1.04**	1.013–1.061	0.97*	0.943–0.997	1.00	0.986–1.022
	(0.010)		(0.012)		(0.014)		(0.009)	
USMLE Step 1	0.99**	0.986–0.997	0.98***	0.973–0.985	0.99**	0.981–0.996	1.02***	1.017–1.028
	(0.003)		(0.003)		(0.004)		(0.003)	
USMLE Step 2	1.01***	1.004–1.013	1.00	0.999–1.010	1.00	0.995–1.008	0.99***	0.984–0.993
	(0.002)		(0.003)		(0.003)		(0.002)	
Work with Underserved	1.01	0.919–1.113	1.08	0.972–1.210	1.13	0.985–1.298	0.86***	0.790–0.937
	(0.049)		(0.061)		(0.080)		(0.037)	
Work/Life Balance	0.89**	0.835–0.956	1.13**	1.047–1.230	0.45***	0.411–0.493	0.87***	0.816–0.927
	(0.031)		(0.046)		(0.021)		(0.028)	
Specialty Personality	0.77***	0.679–0.882	0.94	0.793–1.118	1.20*	1.008–1.441	1.12	0.976–1.276
	(0.052)		(0.083)		(0.110)		(0.076)	
Specialty Competitiveness	0.93	0.863–1.006	0.59***	0.529–0.650	1.05	0.940–1.166	1.17***	1.098–1.252
	(0.036)		(0.031)		(0.057)		(0.039)	
Mentor Advice	1.26***	1.174–1.354	1.06	0.980–1.146	1.04	0.946–1.132	1.01	0.946–1.070
	(0.046)		(0.042)		(0.047)		(0.032)	
Medical School Debt	0.99	0.983–1.000	0.99	0.981–1.001	1.02***	1.009–1.035	1.00	0.992–1.007
	(0.004)		(0.005)		(0.007)		(0.004)	
Publications	0.99	0.978–1.007	1.00	0.980–1.026	0.98	0.951–1.002	1.02***	1.009–1.033
	(0.007)		(0.012)		(0.013)		(0.006)	
Research Experiences	1.05*	1.008–1.095	0.91***	0.857–0.956	0.99	0.931–1.058	1.11***	1.074–1.153
	(0.022)		(0.025)		(0.032)		(0.020)	
Elected to AOA	1.00	0.814–1.238	1.02	0.796–1.318	1.14	0.836–1.566	1.11	0.928–1.321
	(0.107)		(0.132)		(0.183)		(0.100)	
Confidence in Specialty Choice	0.60***	0.542–0.666	0.97	0.842–1.127	0.95	0.805–1.123	1.37***	1.214–1.554
	(0.032)		(0.072)		(0.081)		(0.087)	
Constant	0.87	0.152–5.005	8.32*	1.012–68.47	2.68	0.224–32.02	0.01***	0.00218–0.0522
	(0.777)		(8.949)		(3.390)		(0.009)	
Observations	6,906	6,906	6,906	6,906	6,906	6,906	6,906	6,906

Robust standard errors in parentheses.

*** p<0.001,

** p<0.01,

* p<0.05.

At matriculation, URiM students were more likely to plan for a career in OB/GYN (OR 1.60 95% CI 1.43–1.79) or a surgical field (OR 1.34 95% CI 1.25–1.43) than their non-URiM peers (Table 2). Compared to their non-URiM peers, URiM students were less likely to plan for a career in pediatrics (OR 0.92 95% CI 0.85–0.99) but had no significant difference in their reported interest in IM at matriculation. At the time of graduation, URiM students were less likely to plan to enter pediatrics (OR 0.73 95% CI 0.58–0.93) and more likely to report a planned career in IM (OR 1.26 95% CI 1.04–1.53) compared to non-URiM students (Table 3). No differences in the odds of a planned career in OB/GYN or a surgical field were found between URiM students and non-URiM students at graduation. These results translate to URiM students being 3% more probable to choose IM and 3% less probable to choose pediatrics compared to non-URiM students.

**Fig 2:**
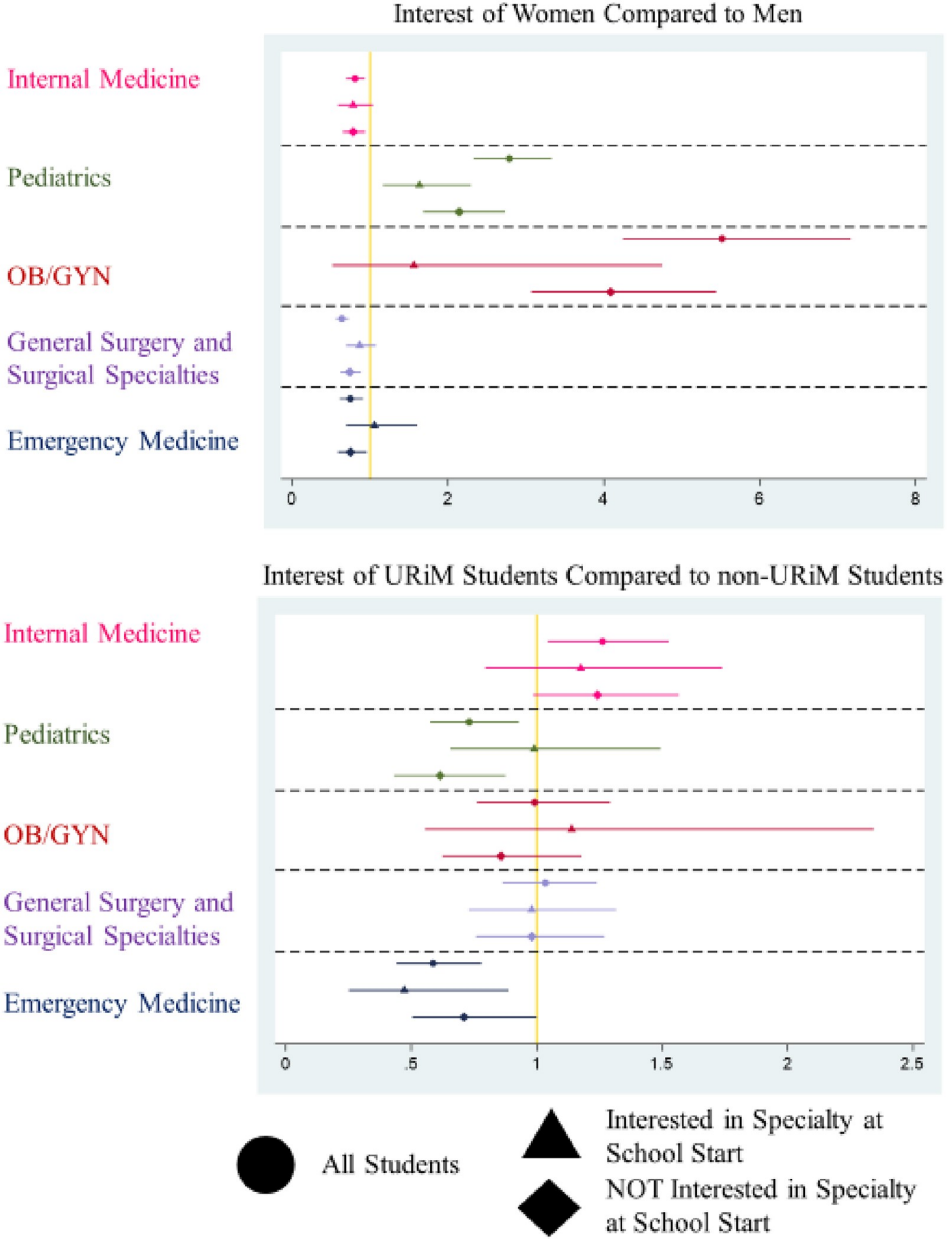
Graduating interest comparison. Plot of Odds Ratios from Three Approaches (All Students regardless of entering interest, Only Students Initially Interested in the specialty at the beginning of Medical School, and Only Students NOT Initially Interested in the specialty at the beginning of Medical School) on a logarithmic scale. X-Axis represents the Odds Ratio of the Outcome in each model for each group. Gold line represents Odds of 1 (no effect). Y-Axis represents the medical specialties investigated. Each point represents the calculated Odds Ratio with the 95% Confidence Interval for each value represented by bars to either side.

Compared to men, women had significantly lower odds of planning for a career in IM or surgery when controlling for their career plans at the start of medical school (Fig 2). In contrast, women were more likely to plan for a career in pediatrics at the time of graduation, even after controlling for their plans at matriculation. Compared to their male peers, women who reported a plan to enter pediatrics at the start of medical school had higher odds to still plan to enter pediatrics at graduation. Female students had higher odds of a planned career in OB/GYN at graduation, even when initial career interest was controlled for in the model. However, women and men exhibited no significant differences in interest in OB/GYN at the time of graduation when controlling for initial interest.

Compared to their peers, URiM students had significantly higher odds of reporting a planned career in IM at the time of graduation, even after controlling for their entering interest in that field (Fig 2). Conversely, even after controlling for their initial specialty selection, URiM students had significantly lower odds of a final career plan in pediatrics upon graduation. When entering career plans were included in the model, no significant difference between URiM and non-URiM students was evident in the other medical specialties (Fig 2).

Several other academic variables (e.g., grades and test scores) and career attitudinal factors were also statistically significantly related to the outcome variables. Their inclusion in the analysis was based on their theoretical importance in decision-making regarding match competitiveness for individuals and a desire to control for these factors on decision making that may confound the direct effects of gender and URiM status on the outcomes of interest. The relative importance of each of these factors on career planning is outside the focus of this analysis, but those interested can find the results in Tables 2 and 3.

## Discussion

The results demonstrate distinctive patterns in medical specialty career plans associated with gender and racial background both in terms of initial career plans at matriculation and the choices made at graduation. Regarding the study hypotheses, while some were supported, the overall patterns were more complex than initially hypothesized. In general, we found that the patterns favoring the proposed under-recruitment mechanism were more often present than those supporting a lack of interest retainment (“cooling out”).

Considering each hypothesis in turn, we found the following. First, specialties with a traditionally higher proportion of female physicians also had higher odds of women initially planning for a career in that field (pediatrics and OB/GYN). Second, two of the four medical specialty groups had statistically different odds of planning to enter a specific specialty for URiM and non-URiM students. Specifically, URiM students had higher odds of reporting a plan to practice in OB/GYN and in a surgical field. This appears to be a new finding as most prior studies did not specifically test correlations with race [71–74]. This may be because prior studies of career plans at matriculation were often smaller in scope [75, 76], older [74, 76–79], and did not include measures of academic competitiveness [71, 79, 80]. Third, changing one’s career path choice appears to be a less common than under-recruitment in terms of explaining preference patterns by gender. Comparatively, women are not being cooled off from a career in fields with relatively fewer female physicians. Instead, it appears a smaller proportionate number of women are becoming interested in those specialties during the course of their medical school careers. Regarding the final hypothesis, URiM student specialty plans were not stable during medical school. Fig 1 indicates where differences in specialty plans between URiM and non-URiM students appear to change between matriculation to medical school and graduation. However, when entering career plans were accounted for, the differences between URiM and non-URiM students’ specialty plans were no longer statistically significant in OB/GYN and surgery (Fig 2). Where changes in planned careers do exist, URiM students have higher odds of choosing IM and lower odds of choosing pediatrics than their peers. In the case of the later, the foremost mechanism we observed was less successful recruitment and not loss of interest by URiM students when compared to their counterparts.

Several limitations of this study must be considered. First, the study uses secondary data and therefore relies on the use of preexisting items which limits the scope of the factors that can be considered. Some variables used are imperfect proxies for underlying constructs, and some factors which could not be considered as they did not exist in the original data. Additionally, privacy policies in place with the primary data holders prohibited the use of some measures, such as the specific medical school attended. Second, the data used is from a period approximately ten years ago and thus the findings may be dated. While this concern cannot be discounted, it is worth noting that applications to each medical specialty have remained largely stable over time as have the relative disparities in representation within each specialty [32, 81]. Where things have changed in the past 5 years, such as decreasing numbers of women applying into EM, this has not resulted in increased representation of women or URiM students. Finally, we were limited to using the eventual specialty of training and not the one in which an applicant may have initially attempted to match. This was as a result of the unwillingness of the National Residency Match Program to share individual level data about applicant match choices, despite requests to gain such access.

In general, this study provides new findings and raises new questions about the underlying mechanisms that result in persistent underrepresentation in some medical specialties. Our results demonstrate that medical specialty choice changes over time and that there are correlations between gender, race, and eventual career aspirations that is missing from the prior empirical work. Differences between specialties, while controlling for academic metrics, suggest that there are structural and cultural effects not currently observed with the data and variables available, and these mechanisms are not currently described in the literature. Our theoretical framework and empirical work provides a road map to better understanding the mechamisms underlying medical choice decisions, yet there remains work to be done.

First, while we attempted to control for some aspects of self-efficacy by including prior academic success, we were unable to account for how prior academic events were interpreted and incorporated into each students self identity. As has been found in work related to persistence through a pre-medical curriculum, men and women may interpret the same grade or test score differently regarding their own underlying abilities [82]. The effect of women perceiving themselves more negatively then men has been shown to be especially true in traditionally male-dominated fields and tasks [83, 84]. Similar mechanisms have been suggested regarding career choice and stereotype threat, sense of belonging, micro-aggressions, and cultural incongruence for both URiM students and women [85–87]. Inaccurate perceptions by students regarding their own ability to succeed in a specific specialty as a result of unwelcoming/non-supportive environments and long term socialization may account for some of the uncontrolled differences observed in our results. Mentorship from physician role models and more transparent peer-to-peer normalization of results may help to ameliorate this effect.

A second potential mechanism derives from our understanding of bounded rationality theory. We used data from only two points in time, the beginning and the end of medical school. BRT suggests decisions such as specialty choice occur over time and are subject to both information limitations and urgency biases. For example, the timing of when students learned about certain specialties and when they were exposed (or not) to physicians from backgrounds similar to theirs is not captured using our design. Studying medical specialty choice in a more granular, and perhaps even prospective manner, that includes sufficient data points for longitudinal modeling could provide a great deal of clarity regarding the decision making process itself in order to inform policy changes in medical education.

Our study suggests that there is no “single solution” to increase the diversity of the physician workforce for all specialties regarding issues of gender or racial underrepresentation. If policy interventions are going to be successful, further study is necessary to describe the mechanisms at work for each individual specialty. We suggest two observed phenomena, loss of interest in a specialty and lower interest generation in a specialty, but understanding the mechanisms operating could be explored either through a qualitative study of students who change their career plans or a quantitative study that uses more detailed temporal data and methods specifically designed to study outcomes that change over time (e.g., fixed effects and/or survival modeling). Our analysis was focused on the four largest specialties in order to establish whether major differences existed between each or if a single pattern could be identified. Future research could focus on those specialties that are considered most competitive or those with the greatest issues regarding representation by using the methods described in this paper.

## Conclusions

When compared with their peers, women and URiM students have differences in their planned specialty of practice that can be identified at the start of medical school for some fields. Worsening underrepresentation in some medical specialties is more likely the result of under-recruitment than loss of initial interest. Further study is necessary to establish the complex mechanisms at work that may be driving changes in career interests over time.
